# Endothelial cell-derived signals in liver development and regeneration

**DOI:** 10.1186/2047-783X-19-S1-S2

**Published:** 2014-06-19

**Authors:** Eckhard Lammert, Jennifer Axnick, Tobias Buschmann

**Affiliations:** 1 Institute of Metabolic Physiology, Heinrich Heine University, 40225 Düsseldorf, Germany; 2 Institute for Beta Cell Biology, German Diabetes Centre, 40225 Düsseldorf, Germany

## 

The liver harbors a specialized capillary bed. It is characterized by Lyve-1 and VEGFR3 expression, low CD31 and vWF expression and lack of CD34 expression [1], Moreover, the basement membrane is discontinuous, and liver sinusoidal endothelial cells are adjacent to a discontinuous basement membrane. Finally, the blood flow through liver sinusoidal capillaries is slow compared to many other organs. Besides the role of hepatic capillary beds in exchange of substances and gases between liver and blood, the endothelium of the liver is required for both liver development [2,3] and liver regeneration [4]. Interestingly, liver regeneration seems to be similar to liver development in that endothelial signals seem to be required during both processes. In the regenerating liver, it was shown that so-called angiocrine signals are needed and involved in discriminating between liver regeneration and liver fibrosis [4,5].

As a model for liver regeneration in mice, a 2/3rd partial hepatectomy is normally performed. Importantly, the liver starts to divide and grow, and within a week the original liver cell mass is restored. Therefore, signals are needed to turn on and off liver regeneration. Previously, Michalopoulos and colleagues have proposed that after PHx three times more blood has to pass the liver when it is reduced to one-third of its original mass based on simple mathematics [6]. To this end, we propose that this increase in blood volume relative to liver mass might turn on angiocrine signals via mechanotransduction (Buschmann, Axnick & Lammert, unpublished data). To this end, we perform different measurements to determine changes in the endothelium, blood flow and blood volume during the course of liver regeneration.
